# Naloxone urban legends and the opioid crisis: what is the role of public health?

**DOI:** 10.1186/s12889-019-7033-5

**Published:** 2019-05-30

**Authors:** Alexis Crabtree, Jeffrey R. Masuda

**Affiliations:** 1 School of Population and Public Health, University of British Columbia, 2206 E Mall, Vancouver, BC V6T 1Z3 Canada; 2 Centre for Environmental Health Equity (CEHE), School of Kinesiology and Health Studies, Queen’s University, 28 Division St KHS301T, Kingston, ON K7L 3N6 Canada

**Keywords:** Naloxone, Opioids, Urban legends

## Abstract

As the overdose crisis in North America continues to deepen, public health leaders find themselves responding to sensational media stories, many of which carry forms and themes that mark them as urban legends.

This article analyzes one set of media accounts – stories of misuse of naloxone, an opioid overdose antidote distributed to people who use drugs – through the lens of social science scholarship on urban legends. We suggest that these stories have met a public need to feel a sense of safety in uncertain times, but function to reinforce societal views of people who use drugs as undeserving of support and resources.

Our field has a duty to speak out in favour of evidence-based programs that support the health of people who use drugs, but the optimal communication strategies are not always clear. Drawing attention to the functions and consequences of urban legends can help frame public health communication in a way that responds to needs without reinforcing prejudices, with application beyond naloxone to the other urban legends that continue to emerge in response to this crisis.

## Background

Frontline emergency responders have alerted the public to a disturbing new trend in the opioid overdose crisis: people are using the syringes in naloxone kits to inject drugs instead of to treat overdoses [1]. Naloxone (also known by its trade name, Narcan**®)** is a medication given to a person who is overdosing in order to reverse opioid-induced respiratory depression. In Canada and the United States, kits containing the medication and syringes to inject it have been distributed to tens of thousands of people at risk of overdose in an effort to provide witnesses with a tool to intervene and save lives [2, 3]. Without the syringes, however, the injectable formulation of the medication is useless. Widespread misuse of the syringes would therefore call into question the efficacy of naloxone kit distribution and raise serious concerns that the funds spent on this intervention (approximately $20 CDN per kit) have been misspent.

Closer examination of this story, however, reveals inconsistencies. For instance, in the community where this story originated, there is a estimated population of 8400 people who use injection drugs, to whom more than 6 million syringes were distributed in 2016 for harm reduction (injection drug) use [4]. Compare this to the mere 4192 naloxone kit syringes distributed in the same area in 2016 and this “supply” argument simply doesn’t make sense. Another practical example is the “delivery” argument: the syringes in naloxone kits are 3 mL each and use a large bore needle for intramuscular injection; they are impractical for injecting drugs intravenously. While it is technically possible for individuals to use naloxone kit syringes to inject drugs, the widespread availability and greater suitability of harm reduction syringes suggests this is almost certainly not a commonly-occurring phenomenon.

Naloxone syringe misuse is not the only prominent media story that can be shown to be highly unlikely. “Yo-yoing”, a term described in media reports that depicts users injecting naloxone alongside opioids to facilitate a greater rush and then subsequent revival [5], is implausible on pharmacological grounds alone: naloxone would block the user’s high entirely. A final example brought to the attention of public health officials in Canada: The widely-cited phenomenon of “Lazarus parties,” described as a scene where people intentionally overdose with the expectation of being resuscitated by naloxone administration, on investigation has been shown to have been an invention of law enforcement, not a phrase or practice in use in the community [6].

These examples reflect a pattern of similar kinds of stories about naloxone circulating with increasing frequency in media reports that consistently fail to reflect the reality of its intended use and demonstrated efficacy in mitigating the harms of the overdose crisis. If this is the case, how are we to make sense of this phenomenon?

## Lessons from URBAN legends

The naloxone stories described above are in many ways consistent with the widely known phenomenon of urban legends. More than just the scary stories recounted to children by over-anxious parents or by teenagers to garner the morbid fascination of their friends, urban legends have been the subject of a substantial amount of scholarship that takes seriously their broader role in society [7, 8]. Pioneering work in this area by sociologists Gerald Best and Gerald Horiuchi [9] has helped us to understand urban legends -- in their case the famous (and thoroughly debunked) story of the razor blade in the Halloween apple reported in newspapers since as early as the 1970s -- as “unconstructed problems,” that are “a product of social strain and of the social organization of the response to that strain” (p. 489). These stories are, in other words, more than stories about specific victims and perpetrators: they are a window into the society that created them. As Best and Horiuchi [9], citing Brunvand [8] describe, urban legends “‘often depict a clash between modern conditions and some aspect of a traditional life-style’...They express fears that the complexities of modern society threaten the traditional social order” (p. 492). In the case of the razor blade, the stories were a reflection of a period of rapid social upheaval in North American society characterized by a fear of increasing crime, mistrust, and individualization.

More pertinent to public health, work by Correll [10] describes the legend of “needle boy” that emerged in the 1980s amidst increasing public awareness -- and fear -- of the HIV pandemic. This urban legend includes stories that depict a range of accounts of covert infection, such as needle attacks occuring in public spaces. Typically beginning with an account of a mysterious feeling of a prick in a public space, it is later revealed that the prick is an HIV-tainted syringe, conveyed back to the victim by through an anonymous and vengeful message such as, “Welcome to the World of AIDS.” These stories often take place in party-like settings known to be associated with salacious behaviour. Correll suggests that these legends represent cautionary tales that warn against those (i.e. the sexually promiscuous) who would transgress the bounds of safe sex. The narrative also works to vilify particular groups (e.g. LGBTQ2S+, sexually objectified racialized women) by portraying them as vengeful disease carriers. Thus, while such legends may serve a useful social purpose of bringing unfamiliar threats into the public consciousness, they may also exacerbate moral panic [11] as well as work to uphold misogynist and racist hegmenonies by putting young, gender non-binary, and minority women (and sometimes men) in their place.

Most recently, Neale and Strang [12] use the concept of “contemporary legends” to explain why people who use drugs describe highly negative experiences with naloxone when it is used in a medical context. They suggest that the medication in this case is acting as a stand-in for societal structures and institutions; attributing negative experiences specifically to naloxone, therefore, allows people who use drugs to share the many difficulties they experience in accessing the health care system (such as stigma and poor medical treatment) and to describe other anxieties about drug use, including withdrawal symptoms and overdose.

Both of these papers highlight how analyzing public narratives as urban legends can deepen public health practitioners’ understandings of emerging phenomena. Most importantly, such sociological analyses suggest underlying issues (fear of the unknown, discrimination) that are areas for engagement in public health messaging.

## Conclusion: what are we to do about naloxone legends?

Taking seriously the stories surrounding the opioid crisis may seem nothing more than a distraction from the real problem at hand -- armchair criticism, or worse, ‘committing sociology’ in a time when urgent response, and the evidence base to justify it, is the only justifiable action. But confronting such stories as the urban legends that they are can help public health leaders to understand why such stories arise and to meet the needs of the people propagating them. In doing so, public health leaders may also play a role in drawing attention to the broader social problems within which the overdose crisis has manifested, including under-funded response systems, the growing housing crisis, and of course, the ongoing discrimination and criminalization of people who use drugs.

A challenge for those working in public health communications in the opioid crisis is formulating how to respond to urban legends in the community. As a field, public health embraces evidence-informed policy and messaging: it can therefore seem natural to counter urban legends with facts or to dismiss them entirely in an effort to attenuate their spread. But, learning from the scholarship on urban legends, we suggest that there is value in identifying the needs that these stories meet for the public and for emergency medical responders. Doing so creates a broader evidence base of sociological knowledge capable of informing a response that addresses and validates these needs.

For the non-drug using public, a key need met by urban legends about naloxone is to make sense of the risks they face by creating a distinction between themselves and the people at highest risk of overdose. The result is to cast people who use drugs as deserving of overdose and death by making them appear irresponsible and unappreciative of efforts directed to help them, and by contrast to frame people who do not use drugs as safe and as deserving of health care resources. The context of the “War on Drugs,” in which drug use is framed as a personal or moral failing rather than a reaction to social conditions, underpins and reinforces this function of urban legends. In a rapidly changing risk environment (i.e. the micro and macro level spaces where physical and social factors interact to produce risk [13]), public health messaging can address the need to feel a sense of certainty and safety – for example, by accurately characterizing the risks associated with prescribed vs illegal opioids [14]. At the same time, the public health response should name and work to counter stigma against people who use drugs through respectful language [15] and by supporting and highlighting the contributions of people who use drugs and their organizations to public health efforts [16–18].

For first responders, from whom some of these legends have reached the media, naloxone urban legends should be interpreted in light of their changing role in the opioid overdose response. With the vastly increased number of overdoses, first responders have been subject to incredible pressures and trauma. Within a system that is so overwhelmed, such stories may very well be a way of making sense of an unsensible scene, or even a cry for help, whether for more resources to increase the front line workforce or for the supports needed to ensure workers are supported when such traumas overwhelm them. As well, the status of naloxone has changed from a prescription medication only used by health professionals, including emergency medical personnel, to one distributed to firefighters, police, librarians, teachers, people who use drugs themselves, and bystanders. The resulting change in scope of practice and in status for first responders as well as the responsibilization of the wider public should therefore be an area of attention in interpreting urban legends, particularly when these stories serve to reinforce the authority of health professionals as the most responsible users of naloxone.

Our call to take urban legends seriously needs especially to be heard from those who put them into the public arena in the first place. For the journalists and reporters mired in a world of clickbait media, the persistent pressure to ‘keep the story going’ feeds a public appetite for ever-more evocative plot twists. Public health has an important role to play in improving media literacy about the complex circumstances that surround the overdose crisis, including the health consequences of further marginalizing drug users.

For user networks, another power of urban legends is to undermine the culture of peer support that has characterized the street response to the epidemic. Public health has the power to humanize the crisis and work to ensure that support groups and activist organizations of people who use drugs are given the social license to operate without political sanction.

To conclude, we offer two modest recommendations for future consideration of the role of urban legends in public health. First, we suggest public health leaders find ways to raise professional awareness of the social function of urban legends and the role that they play in helping listeners to contend with complexity, emotion, and uncertainty. This suggestion may be of particular utility as practitioners contend with urban legends about transdermal fentanyl exposure risks to first responders [19], children [20], and funeral home workers [21]. Second, we echo a long chorus of calls to broaden the knowledge base of evidence-informed public health and ensure that our practitioners draw on the expertise of colleagues in the social sciences and humanities [22], in order to understand the social complexity within which public health operates and better anticipate the unforeseen consequences of our interventions.

## Data Availability

Not applicable
